# Crystal structure of (2,2′-bipyrid­yl)[2,6-bis­(1-butyl-1*H*-benzimidazol-2-yl)pyridine]­chlorido­iridium(III) tri­fluoro­methane­sulfonate

**DOI:** 10.1107/S205698901700010X

**Published:** 2017-01-10

**Authors:** Victoria I. Smith, Mohammad Nozari, Matthias Zeller, Anthony W. Addison

**Affiliations:** a Department of Chemistry, Drexel University, 3141 Chestnut St., Philadelphia, PA, 19104, USA; b Department of Chemistry, Youngstown State University, One University Plaza, Youngstown, OH 44555-3663, USA

**Keywords:** crystal structure, iridium complex, 2,6-bis­(*N*-butyl­benzimidazol-2′-yl)pyridine, 2,2′-bi­pyridine, π–π inter­actions

## Abstract

The title complex compound, [Ir(C_27_H_29_N_5_)Cl(C_10_H_8_N_2_)](CF_3_SO_3_)_2_, was synthesized for a study of iridium(III)/periodate redox systems in water. The coordination geometry of the complex can be best described as distorted octa­hedral, with an r.m.s. deviation of 8.8 (8)% from ideal octa­hedral rectangular geometry. In the crystal, C—H⋯O and C—H⋯F inter­actions between the complex cation and the tri­fluoro­methane­sulfonate anions are observed, as well as a C—H⋯Cl inter­molecular inter­action between neighboring complex cations. In addition, the benzimidazole ring systems display parallel-displaced π–π stacking with centroid–centroid distances of 3.585 (3)–3.907 (3) Å. One of the two tri­fluoro­methane­sulfonate anions is disordered over two orientations with an occupancy ratio of 0.582 (6):0.418 (6). The title complex was characterized using FT–IR, cyclic voltammetry/rotating disc electrode polarography, fluorescence spectrometry, high resolution mass spectrometry, CHN elemental analysis and ^1^H NMR spectroscopy.

## Abstract 2

The title complex, [Ir(C_27_H_29_N_5_)(C_10_H_8_N_2_)Cl]^2+^·2CF_3_SO_3_^−^, was synthesized *via* the reaction of 2,6-bis­(*N*-butyl­benzimidazol-2′-yl)pyridine (bubzimpy) and 2,2′-bi­pyridine (bipy) with sodium hexa­chloro­iridate(III) and precipitated by adding aqueous sodium tri­fluoro­methane­sulfonate solution. The compound was characterized using single-crystal X-ray diffraction, FT–IR, cyclic voltammetry/rotating disc electrode polarography, fluorescence spectrometry, high resolution mass spectrometry, CHN elemental analysis and ^1^H NMR.

## Chemical context   

Some iridium(III) complexes, specifically those containing di­hydroxy­bipyridine ligands, have been shown to catalyze the oxidation of water in the presence of periodate (IO_4_^−^) as the sacrificial oxidant (DePasquale *et al.*, 2013 ▸; Lewandowska-Andralojc *et al.*, 2014 ▸). The title complex was synthesized within a project exploring the nature of iridium(III)/periodate systems in water. The ligands, 2,6-bis­(*N*-butyl­benzimidazol-2′-yl)pyridine (bubzimpy) and 2,2′-bi­pyridine (bipy), were chosen for their denticity characteristics, available donor atoms and solubility characteristics.
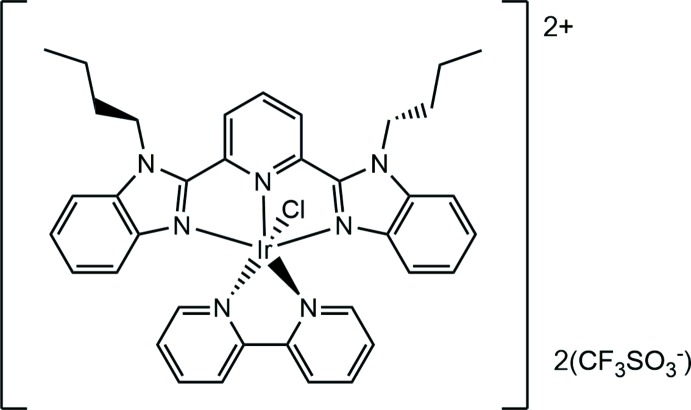


## Structural commentary   

**Figure 1:**
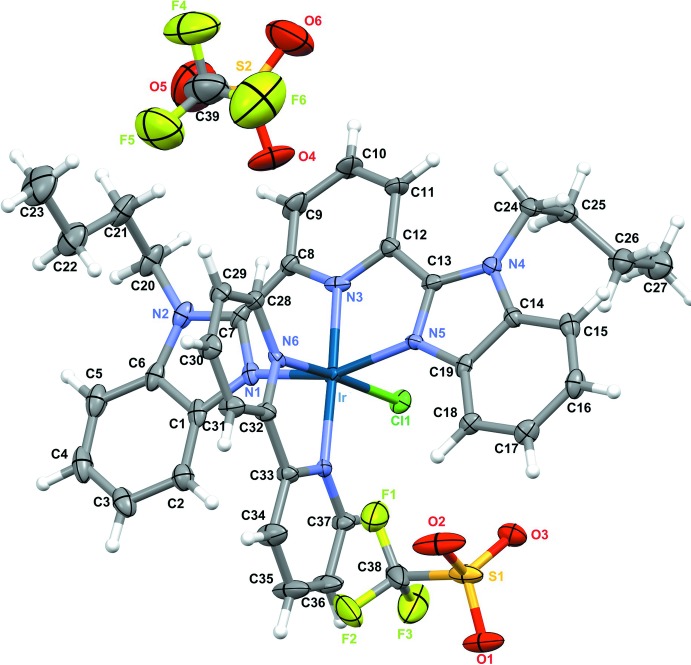
The title complex with two tri­fluoro­methane­sulfonate counter-anions. Displacement ellipsoids are drawn at the 50% probability level. H atoms are rendered as spheres of arbitrary radius. Only one component of the disordered tri­fluoro­methane­sulfonate anion is shown.

The cationic complex of the title salt is composed of one mol­ecule each of bipy and bubzimpy, and a chloride ion coordinating to the iridium(III) atom, with charge balance provided by two crystallographically independent tri­fluoro­methane­sulfonate ions (Fig. 1 ▸). The bond lengths and angles are comparable to similar complexes (Yutaka *et al.*, 2005 ▸), though the torsion angles show distinct differences. The bond angles involving Ir range from 79.55 (12)° (N6—Ir—N7) to 178.09 (13)° (N3—Ir—N7), with the bond lengths between 1.992 (3) Å (Ir—N3) and 2.3510 (9) Å (Ir—Cl). The Ir complex with 2,6-bis­(*N*-methyl­benzimidazol-2′-yl)pyridine (mebzimpy) and bipy synthesized by Yutaka *et al.* (2005 ▸) is closely related to the title complex. Selected bond lengths, bond angles and torsion angles from their complex are compared with those of the title complex in Table 1 ▸. The torsion angle N1—C7—C8—N3 [−6.6 (5)°] for one of the benzimidazoles indicate that the benzimidazole is further removed from coplanarity with the central pyridine plane than it is in the mebzimpy analogue. Meanwhile, the two halves of the coordinating bipy mol­ecule are slightly more rotated *vs* one another than in the mebzimpy analogue, as indicated by the N6—C32—C33—N7 torsion angle of 7.3 (5)°. The dihedral angle between the mean planes of the bubzimpy and bipy ligands is 89.32 (6)°. The r.m.s. angular deviation from ideal octa­hedral rectangularity, defined as 0.312[Σ(θ_*i*_ − 90)^2^]^1/2^ where θ_*i*_ are the twelve *cis*-angles in the pseudo-octa­hedron (Popovitch *et al.*, 2012 ▸), is 8.8 (8)% for the title complex, which is comparable to the value of 7.9 (7)% in the analogous *N*-methyl­ated complex. One of the two tri­fluoro­methane­sulfonate anions in the title complex is disordered over two orientations around the C—S bond with an occupancy ratio of 0.582 (6):0.418 (6).

## Supra­molecular features   

**Figure 2:**
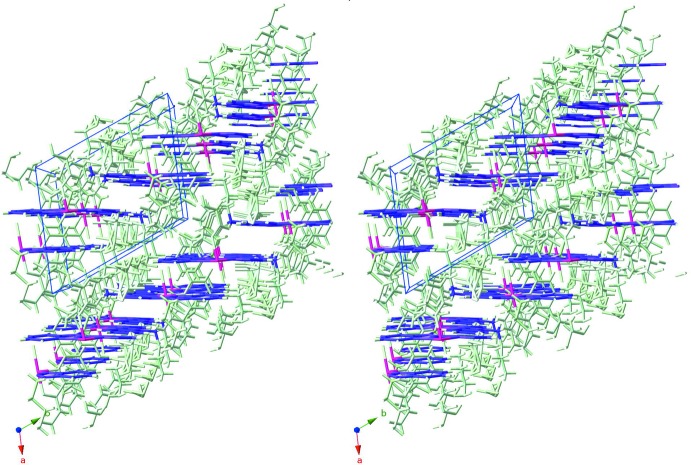
A perspective view (from 150 Å, inverse stereo stick-structure) along the *c*-axis direction, with the bis­(benzimidazol­yl)pyridine-Ir planes oriented horizontally and rendered in purple, *versus* the other atoms (pale green). The slipped stacks form a ‘staircase’; in the *N*-methyl analogue (Yutaka *et al.*, 2005 ▸), the corresponding array appears as an alternating ‘stepping stone’ pattern.

**Figure 3:**
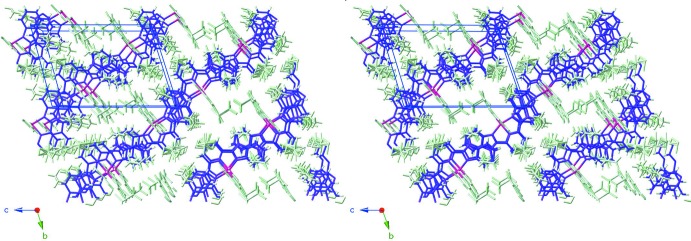
Similarly to Fig. 2 ▸, a view (inverse stereo stick-structure) along the *a*-axis direction, showing the bis­(benzimidazol­yl)pyridines (purple) and the other atoms (pale green).

The mol­ecules stack in the crystal so that the benzimidazole ring systems of neighbouring mol­ecules are parallel to each other, enabling π–π inter­actions to occur. The centroid–centroid distances and the slippages of the slipped π–π stacking inter­actions are given in Table 2 ▸. The shortest inter­planar distance is 3.337 (6) Å with the two π–π stacked benzene rings slipped by 2.033 (8) Å. These inter­actions link the mol­ecules into a staircase structure along [011] as shown in Figs. 2 ▸ and 3 ▸. The slipped π–π stacking arrangement (Fig. 3 ▸) suggests that isomorphous replacement of iridium(III) mol­ecules by non-luminescent/non-quenching analogues could lead to the formation of a superantenna system (Mikhalyova *et al.*, 2015 ▸). The two distinct tri­fluoro­methane­sulfonate anions balance the complex charge and display C—H⋯O and C—H⋯F hydrogen bonds (Table 3 ▸). These inter­actions involve the O and F atoms from the anions inter­acting with the CH units from bipy as well as the pyridine ring of bubzimpy. An inter­molecular C—H⋯Cl inter­action is also observed between the coordinating chloride ion and the benzimidazole ring of bubzimpy on the neighboring complex (Table 3 ▸). Although this inter­action is weaker than the prominent C—H⋯O inter­actions, it contributes to the overall orientation of the packing in the crystal.

## Electrochemistry   

The redox chemistry of the Ir^III^ complex was studied using cyclic voltammetry (CV) and rotating disc electrode (RDE) polarography, which were performed at 298 K on 0.3 m*M* Ir complex in aceto­nitrile with 0.1 *M* tetra­butyl­ammonium hexa­fluorido­phosphate (TBAPF_6_) as the supporting electrolyte, at scan rates ranging from 50 to 800 mV s^−1^ for CV, and 1200 and 2400 rpm for the RDE. Experiments were run on a BASi-Epsilon instrument using a three-electrode cell, a non-aqueous reference electrode (APE) (Pavlishchuk & Addison, 2000 ▸) and a 3 mm diameter Pt disc working electrode. No well-defined anodic process is observed below +1400 mV, indicating that the oxidative potential for the Ir complex is higher than the potential window available in our experiments. The cathodic electrochemistry is not straightforward; however, there are three reductive processes with cathodic peak potentials of −1211, −1472 and −1719 mV. Similar results have been reported for the mebzimpy complex (Yutaka *et al.*, 2005 ▸). In the RDE polarogram, a reductive wave was seen at *E*_1/2_ = −1042±5 mV, from which the diffusion coefficient of the mol­ecule is estimated to be *D* = 9.0×10^−6^ cm^2^ s^−1^ in MeCN, corresponding to a *Dη* value of 3.3×10 ^−8^ g cm s^−2^, consistent with a one-electron transfer.

## UV–Vis and Fluorimetry   

**Figure 4:**
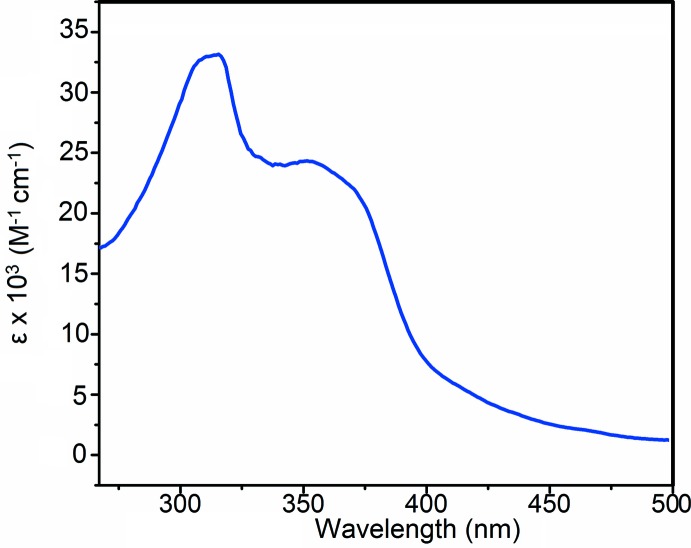
UV–Vis spectrum of the title complex (10 µ*M*) in aceto­nitrile.

**Figure 5:**
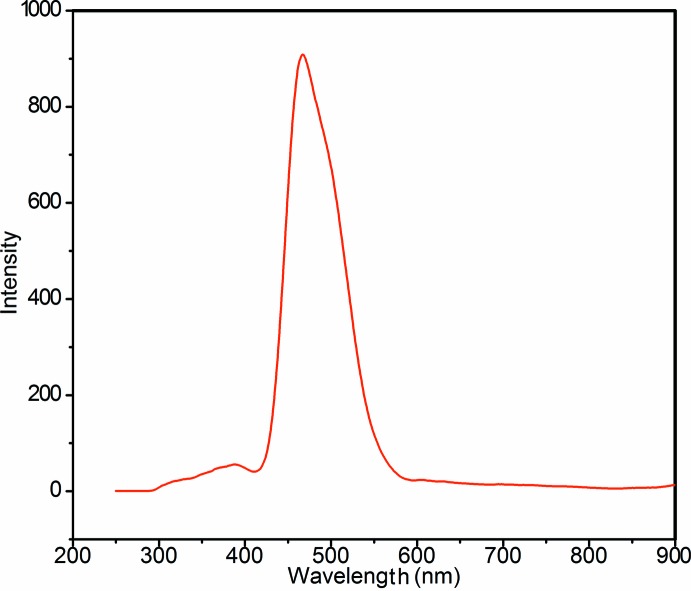
Emission spectrum of the title Ir(III) complex (0.8 µ*M*) in non-purged aceto­nitrile at ambient temperature, excited at 295 nm. The ordinate unit is arbitrary.

The photochemical and photophysical properties of iridium(III) complexes have been studied extensively in the last few decades in order to better understand their potential for applications in areas like solar energy and electroluminescence (EL) devices (Nazeeruddin *et al.*, 2003 ▸ *).* The optical absorption spectrum of the title complex is displayed in Fig. 4 ▸. In such mixed-ligand complexes, ligand π–π* transition bands typically overlap; however, the ligand π–π* bands for bipy and bubzimpy in our complex were well-resolved at 315 and 352 nm, respectively, similarly to those observed by Yutaka *et al.* (2005 ▸). As has often been observed in compounds of this type (Yutaka *et al.*, 2005 ▸), there is a strong emission in the yellow region of the spectrum with the intensity peaking at 542 nm (Fig. 5 ▸). The excitation profile is dominated by an absorption maximizing at 302 nm, corresponding closely to the bipy π–π* transition at 315 nm.

## Database survey   

Crystal structures of complexes containing bubzimpy as a ligand exist in the literature. This ligand chelates well to other transition metals, such as ruthenium (Yu *et al.*, 2012 ▸), copper (Kose *et al.*, 2014 ▸), gadolinium, lanthanum (Drew *et al.*, 2004 ▸) and manganese (Kose & McKee, 2014 ▸). Hijazi *et al.* (2010 ▸) reported a platinum complex with a ligand similar to bubzimpy, 2,6-di(*N*-hexyl­benzimidazol-2′-yl)pyridine. Similarly, Mathew & Sun (2010 ▸) showed a variety of 2,6-bis­(*N*-alkyl­benzimidazol-2′-y)pyridine platinum(II) complexes with one coordinating chloride as in our iridium complex. These platinum complexes involved variation of the alkyl chain on the benzimidazole ligand, as well as varied counter-ions, such as PF_6_^−^, ClO_4_^−^, and BF_4_^−^.

## Synthesis and crystallization   

**Figure 6:**
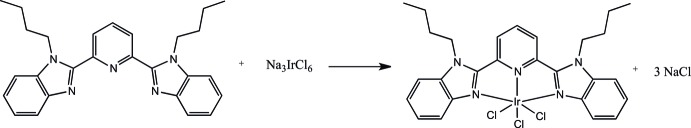
Step 1: Reaction of bubzimpy with hexa­chlorido­iridate(III) in a 1:1 ratio.

**Figure 7:**
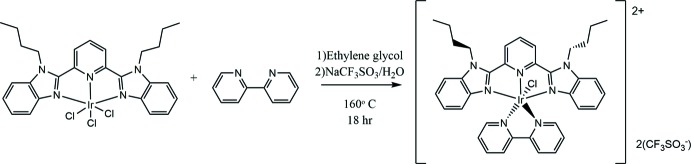
Step 2: Reaction of [2,6-bis-(*N*-butyl­benzimidazol-2′-yl)pyridine]­tri­chlorido­iridium(III) with bipy.

The bubzimpy ligand used was prepared using a previously reported alkyl­ation method (Nozari *et al.*, 2014 ▸). The title complex was synthesized following a method adapted from the literature (Yutaka *et al.*, 2005 ▸)*.* Sodium hexa­chlorido­iridate(IV) (0.28 g, 0.5 mmol) was reduced to hexa­chlorido­iridate(III) with ascorbic acid under a nitro­gen atmosphere. The reduced iridium and the bubzimpy (0.36 g, 0.5 mmol) were dissolved in warm ethyl­ene glycol (5 mL) and then heated on a steam bath for 4 h, after which the reddish brown solid was filtered off and washed with ether and chloro­form (Fig. 6 ▸). This resulting trichlorido-inter­mediate [0.057 g, 78 mmol; FAB-LSIMS MS: calculated (*m*+) *m*/*z* 721.110, found 721.135] was then dissolved in hot ethyl­ene glycol (10 mL) with 2,2′-bi­pyridine (0.015 g, 94 mmol) and stirred at 433 K for 18 h (Fig. 7 ▸). The resulting iridium complex was precipitated by addition of aqueous sodium tri­fluoro­methane­sulfonate and then filtered off and washed with ether and chloro­form. The crude product was purified *via* a two month diffusion of toluene into a methyl­ene chloride solution, yielding orange crystals. M.p. > 523 K; Analysis calculated: C 42.3, H 3.35, N 8.86; found: C 42.7, H 3.70, N 9.06; ^1^H NMR (500 MHz, C_2_D_6_OS): δ 10.1 (*d*, 1H), 9.20 (*d*, 1H), 8.90 (*d*, 1H), 8.82 (*d*, 1H), 8.75–8.67(*t*, 2H), 8.43 (*t*, 1H),8.13 (*m*, 1H), 8.07 (*m*, 1H), 7.94 (*m*, 2H), 7.72 (*t*, 1H), 7.59 (*m*, 2H), 7.49 (*t*, 1H), 7.30 (*m*, 2H), 5.90 (*m*, 2H), 3.41 (*m*, 4H), 1.95 (*m*, 4H), 1.49–1.35 (*m*, 4H), 0.99–0.74 (*m*, 6H); FT–IR: 3085, 2959, 2873, 1606, 1466, 1451, 1154, 844, 745 cm^−1^; FAB MS: calculated (*m*-CF_3_SO_3_)^+^ *m*/*z* 956.195, found 956.198.

## Refinement   

Crystal data, data collection and structure refinement details are summarized in Table 4 ▸. H atoms were positioned geometrically and constrained to ride on their parent atoms, with C—H bond lengths of 0.95, 0.99 and 0.98 Å for aromatic CH, aliphatic CH_2_ and CH_3_ groups, respectively. Methyl H atoms were allowed to rotate but not to tip to best fit the experimental electron density. *U*_iso_(H) values were set to a multiple of *U*_eq_(C) with 1.5 for CH_3_ and 1.2 for CH and CH_2_ units.

One of the two tri­fluoro­methane­sulfonate anions was refined as disordered over two orientations [occupancy ratio 0.582 (6):0.418 (6)]. The two components were restrained to have geometries similar to that of the non-disordered anion (*SAME* with esd 0.02 Å), and the disordered atoms were subjected to a rigid-bond restraint (*RIGU* with esd 0.001 Å^2^). Reflections 001 and 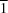
10 affected by the beam stop were omitted from the refinement. The residual electron density peaks of 3.18 and 3.12 e Å^−3^ are located 0.89 and 0.85 Å, respectively, from atom Ir.

## Supplementary Material

Crystal structure: contains datablock(s) I, Global. DOI: 10.1107/S205698901700010X/is5464sup1.cif

Structure factors: contains datablock(s) I. DOI: 10.1107/S205698901700010X/is5464Isup2.hkl

CCDC reference: 1525487

Additional supporting information:  crystallographic information; 3D view; checkCIF report

## Figures and Tables

**Table 1:** Comparison of selected bond lengths, bond angles and torsion angles (Å, °)

	(bipy)(mebzimpy)chlorido­iridium(III)(PF_6_)_2_ (Yutaka *et al.*, 2005 ▸) (geometry: slightly distorted octa­hedral)	Title complex (geometry: slightly distorted octa­hedral)
Bond Length		
Ir—Cl	2.338 (3)	2.3510 (9)
Ir—N1	2.039 (8)	2.032 (3)
Ir—N3	1.991 (8)	1.992 (3)
Ir—N5	2.032 (9)	2.037 (3)
Ir—N6	2.046 (9)	2.050 (3)
Ir—N7	2.049 (9)	2.057 (3)
		
Bond Angles		
N3—Ir—N5	78.9 (3)	80.34 (13)
N3—Ir—N7	178.5 (4)	178.09 (13)
N6—Ir—N7	81.0 (4)	79.55 (12)
N1—Ir—N5	156.3 (3)	158.99 (13)
N3—Ir—N6	103.4 (2)	99.62 (12)
		
Torsion Angles		
N1—C7—C8—N3	0 (1)	−6.6 (5)
N3—C12—C13—N5	−1 (1)	−1.1 (5)
N6—C32—C33—N7	4 (1)	7.3 (5)

Atom labels correspond to atoms of the title complex, analogous relationships reported by Yutaka *et al.* (2005 ▸) were compared.

**Table 2:** π–π inter­actions (Å) with centroid–centroid distances less than 4 Å *Cg*4, *Cg*5,*Cg*9 and *Cg*10 are the centroids of the N1/C1/C6/N2/C7, N4/C13/N5/C19/C14, C1–C6 and C14–C19 rings, respectively.

*Cg*(*I*)⋯*Cg*(*J*)	*Cg*⋯*Cg* distance	Slippage
*Cg*4⋯*Cg*9^i^	3.596 (3)	1.204
*Cg*5⋯*Cg*10^iii^	3.585 (3)	1.311
*Cg*10⋯*Cg*10^iii^	3.907 (3)	2.033

Symmetry codes: (i) −*x* + 1, −*y*, −*z*; (iii) −*x* + 1, −*y* + 1, −*z* + 1.

**Table 3:** Hydrogen-bond geometry (Å, °)

*D*—H⋯*A*	*D*—H	H⋯*A*	*D*⋯*A*	*D*—H⋯*A*
C5—H5⋯Cl1^i^	0.95	2.74	3.422 (4)	130
C9—H9⋯O5^ii^	0.95	2.42	3.084 (11)	126
C9—H9⋯O5*B* ^ii^	0.95	2.19	3.052 (13)	151
C20—H20*B*⋯O6^ii^	0.99	2.48	3.259 (13)	135
C20—H20*B*⋯O5*B* ^ii^	0.99	2.52	3.406 (13)	149
C24—H24*B*⋯O3^iii^	0.99	2.46	3.419 (5)	163
C25—H25*A*⋯F2^iv^	0.99	2.56	3.287 (5)	131
C28—H28⋯O4	0.95	2.19	3.063 (11)	152
C28—H28⋯O4*B*	0.95	2.34	3.196 (18)	150
C31—H31⋯O2^v^	0.95	2.45	3.380 (5)	165
C34—H34⋯O2^v^	0.95	2.35	3.298 (5)	177
C36—H36⋯O3^vi^	0.95	2.45	3.333 (5)	155
C37—H37⋯O1^vi^	0.95	2.49	3.302 (5)	144

Symmetry codes: (i) 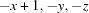; (ii) 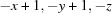; (iii) 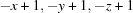; (iv) 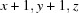; (v) 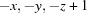; (vi) 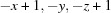.

**Table 4:** Experimental details

Crystal data	
Chemical formula	[Ir(C_27_H_29_N_5_)Cl(C_10_H_8_N_2_)](CF_3_O_3_S)_2_
*M*_r_	1105.52
Crystal system, space group	Triclinic, *P* 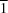
Temperature (K)	100
*a*, *b*, *c* (Å)	10.7731 (6), 13.1932 (6), 17.0021 (9)
α, β, γ (°)	104.530 (2), 96.3822 (16), 110.8357 (15)
*V* (Å^3^)	2131.96 (19)
*Z*	2
Radiation type	Mo *K*α
μ (mm^−1^)	3.37
Crystal size (mm)	0.21 × 0.11 × 0.09

Data collection	
Diffractometer	Bruker AXS D8 Quest CMOS diffractometer
Absorption correction	Multi-scan (*SADABS*; Bruker, 2014 ▸)
*T*_min_, *T*_max_	0.580, 0.746
No. of measured, independent and observed [*I* > 2σ(*I*)] reflections	32148, 12026, 9498
*R*_int_	0.048
(sin θ/λ)_max_ (Å^−1^)	0.715

Refinement	
*R*[*F* ^2^ > 2σ(*F* ^2^)], *wR*(*F* ^2^), *S*	0.042, 0.081, 1.03
No. of reflections	12026
No. of parameters	634
No. of restraints	171
H-atom treatment	H-atom parameters constrained
Δρ_max_, Δρ_min_ (e Å^−3^)	3.37, −1.91

Computer programs: *APEX2* and *SAINT* (Bruker, 2014 ▸), *SHELXS97* (Sheldrick, 2008 ▸), *SHELXL-2014/7* (Sheldrick, 2015 ▸) and *SHELXLE* (Hübschle *et al.*, 2011 ▸).

## supplementary crystallographic information

### Crystal data

**Table:**

[Ir(C_27_H_29_N_5_)Cl(C_10_H_8_N_2_)](CF_3_O_3_S)_2_	*Z* = 2
*M**_r_* = 1105.52	*F*(000) = 1096
Triclinic, *P*1	*D*_x_ = 1.722 Mg m^−^^3^
*a* = 10.7731 (6) Å	Mo *K*α radiation, λ = 0.71073 Å
*b* = 13.1932 (6) Å	Cell parameters from 9841 reflections
*c* = 17.0021 (9) Å	θ = 2.4–30.5°
α = 104.530 (2)°	µ = 3.37 mm^−^^1^
β = 96.3822 (16)°	*T* = 100 K
γ = 110.8357 (15)°	Block, orange
*V* = 2131.96 (19) Å^3^	0.21 × 0.11 × 0.09 mm

### Data collection

**Table:**

Bruker AXS D8 Quest CMOS diffractometer	12026 independent reflections
Radiation source: I-mu-S microsource X-ray tube	9498 reflections with *I* > 2σ(*I*)
Laterally graded multilayer (Goebel) mirror monochromator	*R*_int_ = 0.048
ω and phi scans	θ_max_ = 30.5°, θ_min_ = 2.2°
Absorption correction: multi-scan (SADABS; Bruker, 2014)	*h* = −15→15
*T*_min_ = 0.580, *T*_max_ = 0.746	*k* = −17→18
32148 measured reflections	*l* = −21→24

### Refinement

**Table:**

Refinement on *F*^2^	Primary atom site location: structure-invariant direct methods
Least-squares matrix: full	Secondary atom site location: difference Fourier map
*R*[*F*^2^ > 2σ(*F*^2^)] = 0.042	Hydrogen site location: inferred from neighbouring sites
*wR*(*F*^2^) = 0.081	H-atom parameters constrained
*S* = 1.03	*w* = 1/[σ^2^(*F*_o_^2^) + (0.0341*P*)^2^] where *P* = (*F*_o_^2^ + 2*F*_c_^2^)/3
12026 reflections	(Δ/σ)_max_ = 0.001
634 parameters	Δρ_max_ = 3.37 e Å^−^^3^
171 restraints	Δρ_min_ = −1.91 e Å^−^^3^

### Special details

**Table:**

Geometry. All esds (except the esd in the dihedral angle between two l.s. planes) are estimated using the full covariance matrix. The cell esds are taken into account individually in the estimation of esds in distances, angles and torsion angles; correlations between esds in cell parameters are only used when they are defined by crystal symmetry. An approximate (isotropic) treatment of cell esds is used for estimating esds involving l.s. planes.
Refinement. One of the two triflate anions is disordered with two alternative orientations. The two moieties were restrained to geometries similar to that of the not disordered anion, and disordered atoms were subjected to a rigid bond restraint (RIGU in Shelxl). Reflections 0 0 1 and -1 1 0 were affected by the beam stop and were omitted from the refinement.

### Fractional atomic coordinates and isotropic or equivalent isotropic displacement parameters (Å^2^)

**Table:**

	*x*	*y*	*z*	*U*_iso_*/*U*_eq_	Occ. (<1)
C1	0.3587 (4)	0.0118 (3)	0.0770 (3)	0.0220 (9)	
C2	0.3051 (4)	−0.0916 (3)	0.0931 (3)	0.0256 (9)	
H2	0.3113	−0.0954	0.1483	0.031*	
C3	0.2418 (4)	−0.1890 (4)	0.0243 (3)	0.0324 (11)	
H3	0.2026	−0.2614	0.0324	0.039*	
C4	0.2347 (5)	−0.1824 (4)	−0.0572 (3)	0.0370 (12)	
H4	0.1916	−0.2510	−0.1026	0.044*	
C5	0.2878 (4)	−0.0805 (4)	−0.0738 (3)	0.0330 (11)	
H5	0.2819	−0.0769	−0.1291	0.040*	
C6	0.3503 (4)	0.0167 (4)	−0.0050 (2)	0.0245 (9)	
C7	0.4573 (4)	0.1915 (3)	0.0822 (2)	0.0191 (8)	
C8	0.5313 (4)	0.3152 (3)	0.1233 (2)	0.0193 (8)	
C9	0.5901 (4)	0.4009 (4)	0.0896 (3)	0.0313 (10)	
H9	0.5826	0.3844	0.0311	0.038*	
C10	0.6605 (5)	0.5118 (4)	0.1431 (3)	0.0354 (11)	
H10	0.7022	0.5715	0.1208	0.043*	
C11	0.6711 (4)	0.5371 (4)	0.2287 (3)	0.0279 (10)	
H11	0.7175	0.6135	0.2646	0.033*	
C12	0.6128 (4)	0.4491 (3)	0.2604 (3)	0.0211 (8)	
C13	0.6136 (4)	0.4471 (3)	0.3461 (2)	0.0185 (8)	
C14	0.6482 (4)	0.4872 (3)	0.4835 (2)	0.0176 (8)	
C15	0.6864 (4)	0.5366 (3)	0.5698 (3)	0.0230 (9)	
H15	0.7398	0.6159	0.5953	0.028*	
C16	0.6425 (4)	0.4645 (4)	0.6160 (3)	0.0255 (9)	
H16	0.6670	0.4951	0.6751	0.031*	
C17	0.5626 (4)	0.3468 (3)	0.5792 (3)	0.0234 (9)	
H17	0.5343	0.3004	0.6138	0.028*	
C18	0.5246 (4)	0.2976 (3)	0.4937 (2)	0.0184 (8)	
H18	0.4709	0.2183	0.4686	0.022*	
C19	0.5683 (4)	0.3693 (3)	0.4460 (2)	0.0175 (8)	
C20	0.4182 (5)	0.1735 (4)	−0.0728 (3)	0.0327 (11)	
H20A	0.4005	0.1097	−0.1238	0.039*	
H20B	0.5107	0.2318	−0.0657	0.039*	
C21	0.3146 (5)	0.2257 (4)	−0.0839 (3)	0.0367 (11)	
H21A	0.3215	0.2515	−0.1338	0.044*	
H21B	0.3376	0.2937	−0.0350	0.044*	
C22	0.1683 (5)	0.1437 (5)	−0.0938 (4)	0.0451 (13)	
H22A	0.1574	0.1257	−0.0411	0.054*	
H22B	0.1480	0.0715	−0.1383	0.054*	
C23	0.0683 (6)	0.1953 (6)	−0.1156 (4)	0.0654 (18)	
H23A	0.0768	0.2105	−0.1687	0.098*	
H23B	0.0885	0.2668	−0.0717	0.098*	
H23C	−0.0248	0.1416	−0.1205	0.098*	
C24	0.7624 (4)	0.6541 (3)	0.4311 (3)	0.0243 (9)	
H24A	0.7648	0.7019	0.4868	0.029*	
H24B	0.7227	0.6803	0.3888	0.029*	
C25	0.9067 (4)	0.6697 (3)	0.4235 (3)	0.0275 (9)	
H25A	0.9558	0.7474	0.4205	0.033*	
H25B	0.9031	0.6143	0.3709	0.033*	
C26	0.9862 (4)	0.6534 (4)	0.4960 (3)	0.0332 (11)	
H26A	0.9386	0.5752	0.4986	0.040*	
H26B	0.9892	0.7080	0.5489	0.040*	
C27	1.1315 (5)	0.6718 (4)	0.4868 (4)	0.0421 (12)	
H27A	1.1288	0.6200	0.4334	0.063*	
H27B	1.1778	0.6562	0.5325	0.063*	
H27C	1.1810	0.7510	0.4886	0.063*	
C28	0.2264 (4)	0.2617 (3)	0.2236 (2)	0.0182 (8)	
H28	0.2797	0.3178	0.2016	0.022*	
C29	0.0938 (4)	0.2494 (3)	0.2276 (3)	0.0234 (9)	
H29	0.0567	0.2964	0.2083	0.028*	
C30	0.0167 (4)	0.1683 (3)	0.2598 (3)	0.0261 (9)	
H30	−0.0733	0.1599	0.2642	0.031*	
C31	0.0723 (4)	0.0993 (3)	0.2858 (3)	0.0257 (9)	
H31	0.0201	0.0422	0.3074	0.031*	
C32	0.2048 (4)	0.1139 (3)	0.2801 (2)	0.0183 (8)	
C33	0.2706 (4)	0.0411 (3)	0.3017 (2)	0.0189 (8)	
C34	0.2044 (4)	−0.0575 (4)	0.3209 (3)	0.0340 (11)	
H34	0.1119	−0.0794	0.3252	0.041*	
C35	0.2739 (5)	−0.1238 (4)	0.3338 (4)	0.0455 (14)	
H35	0.2292	−0.1924	0.3461	0.055*	
C36	0.4088 (5)	−0.0899 (4)	0.3288 (3)	0.0367 (12)	
H36	0.4576	−0.1352	0.3370	0.044*	
C37	0.4723 (4)	0.0113 (3)	0.3117 (3)	0.0218 (8)	
H37	0.5659	0.0360	0.3098	0.026*	
Cl1	0.70261 (9)	0.22427 (8)	0.26931 (6)	0.0210 (2)	
Ir	0.47773 (2)	0.21221 (2)	0.25420 (2)	0.01354 (5)	
N1	0.4271 (3)	0.1240 (3)	0.1307 (2)	0.0178 (7)	
N2	0.4136 (3)	0.1306 (3)	0.0000 (2)	0.0236 (8)	
N3	0.5444 (3)	0.3419 (3)	0.2083 (2)	0.0176 (7)	
N4	0.6751 (3)	0.5341 (3)	0.4193 (2)	0.0189 (7)	
N5	0.5502 (3)	0.3484 (2)	0.36020 (19)	0.0149 (6)	
N6	0.2805 (3)	0.1961 (2)	0.25020 (18)	0.0127 (6)	
N7	0.4044 (3)	0.0751 (2)	0.29777 (19)	0.0148 (6)	
S1	0.23767 (11)	0.10966 (9)	0.66797 (8)	0.0310 (3)	
O1	0.2471 (3)	0.0300 (3)	0.7107 (2)	0.0433 (9)	
O2	0.1201 (4)	0.1376 (3)	0.6721 (3)	0.0604 (12)	
O3	0.3621 (3)	0.2058 (2)	0.67949 (19)	0.0302 (7)	
C38	0.2054 (5)	0.0301 (4)	0.5598 (4)	0.0485 (15)	
F1	0.1975 (4)	0.0920 (3)	0.5085 (2)	0.0599 (10)	
F2	0.0911 (4)	−0.0634 (3)	0.5361 (3)	0.1020 (18)	
F3	0.3068 (4)	−0.0043 (3)	0.5431 (2)	0.0668 (11)	
S2	0.2918 (3)	0.5497 (3)	0.1313 (2)	0.0543 (10)	0.582 (6)
O4	0.3281 (12)	0.4757 (10)	0.1688 (7)	0.058 (3)	0.582 (6)
O5	0.2739 (13)	0.4969 (10)	0.0387 (5)	0.118 (4)	0.582 (6)
O6	0.3639 (11)	0.6683 (7)	0.1562 (9)	0.133 (5)	0.582 (6)
C39	0.1205 (10)	0.5273 (10)	0.1400 (8)	0.076 (2)	0.582 (6)
F4	0.0816 (9)	0.6038 (8)	0.1201 (8)	0.111 (3)	0.582 (6)
F5	0.0356 (8)	0.4249 (6)	0.1081 (7)	0.107 (3)	0.582 (6)
F6	0.1377 (13)	0.5693 (12)	0.2280 (6)	0.138 (4)	0.582 (6)
S2B	0.2732 (5)	0.4888 (5)	0.1166 (3)	0.0588 (14)	0.418 (6)
O4B	0.358 (2)	0.5038 (15)	0.1939 (9)	0.072 (5)	0.418 (6)
O5B	0.3405 (17)	0.5776 (12)	0.0763 (10)	0.110 (5)	0.418 (6)
O6B	0.1775 (17)	0.3759 (9)	0.0761 (9)	0.132 (6)	0.418 (6)
C39B	0.1601 (16)	0.5559 (13)	0.1526 (11)	0.095 (4)	0.418 (6)
F4B	0.0708 (19)	0.5400 (16)	0.0816 (10)	0.159 (6)	0.418 (6)
F5B	0.2410 (17)	0.6581 (11)	0.2000 (10)	0.159 (6)	0.418 (6)
F6B	0.0723 (17)	0.4905 (13)	0.1949 (12)	0.127 (5)	0.418 (6)

### Atomic displacement parameters (Å^2^)

**Table:**

	*U*^11^	*U*^22^	*U*^33^	*U*^12^	*U*^13^	*U*^23^
C1	0.0131 (18)	0.029 (2)	0.021 (2)	0.0120 (16)	0.0014 (15)	−0.0016 (18)
C2	0.021 (2)	0.028 (2)	0.026 (2)	0.0095 (17)	0.0038 (17)	0.0046 (18)
C3	0.026 (2)	0.026 (2)	0.037 (3)	0.0123 (18)	0.0028 (19)	−0.003 (2)
C4	0.028 (2)	0.040 (3)	0.030 (3)	0.017 (2)	−0.0037 (19)	−0.012 (2)
C5	0.030 (2)	0.045 (3)	0.021 (2)	0.022 (2)	−0.0003 (18)	−0.003 (2)
C6	0.018 (2)	0.038 (2)	0.019 (2)	0.0163 (18)	0.0022 (16)	0.0049 (18)
C7	0.0186 (19)	0.031 (2)	0.0123 (18)	0.0133 (16)	0.0040 (15)	0.0092 (16)
C8	0.0174 (19)	0.024 (2)	0.0179 (19)	0.0075 (15)	0.0033 (15)	0.0097 (16)
C9	0.024 (2)	0.047 (3)	0.027 (2)	0.009 (2)	0.0074 (18)	0.025 (2)
C10	0.034 (3)	0.035 (3)	0.034 (3)	0.000 (2)	0.003 (2)	0.027 (2)
C11	0.029 (2)	0.023 (2)	0.029 (2)	0.0045 (17)	0.0000 (18)	0.0163 (19)
C12	0.0170 (19)	0.0185 (19)	0.028 (2)	0.0062 (15)	0.0012 (16)	0.0098 (17)
C13	0.0173 (19)	0.0190 (19)	0.024 (2)	0.0084 (15)	0.0048 (15)	0.0119 (17)
C14	0.0161 (18)	0.0161 (18)	0.0190 (19)	0.0063 (14)	0.0030 (15)	0.0035 (16)
C15	0.0179 (19)	0.022 (2)	0.024 (2)	0.0094 (16)	0.0016 (16)	−0.0013 (17)
C16	0.023 (2)	0.034 (2)	0.017 (2)	0.0132 (18)	0.0036 (16)	0.0010 (18)
C17	0.021 (2)	0.032 (2)	0.022 (2)	0.0118 (17)	0.0098 (16)	0.0118 (18)
C18	0.0163 (18)	0.025 (2)	0.0184 (19)	0.0097 (15)	0.0077 (15)	0.0101 (16)
C19	0.0121 (17)	0.025 (2)	0.0171 (19)	0.0101 (15)	0.0015 (14)	0.0057 (16)
C20	0.033 (2)	0.056 (3)	0.017 (2)	0.024 (2)	0.0081 (18)	0.014 (2)
C21	0.032 (3)	0.059 (3)	0.026 (2)	0.023 (2)	0.004 (2)	0.020 (2)
C22	0.031 (3)	0.060 (3)	0.049 (3)	0.021 (2)	0.006 (2)	0.022 (3)
C23	0.034 (3)	0.088 (5)	0.082 (5)	0.030 (3)	0.004 (3)	0.035 (4)
C24	0.023 (2)	0.0147 (18)	0.033 (2)	0.0068 (15)	−0.0006 (17)	0.0069 (18)
C25	0.023 (2)	0.019 (2)	0.038 (3)	0.0060 (16)	0.0020 (18)	0.0112 (19)
C26	0.022 (2)	0.033 (2)	0.046 (3)	0.0110 (18)	0.004 (2)	0.016 (2)
C27	0.027 (2)	0.043 (3)	0.061 (4)	0.016 (2)	0.009 (2)	0.021 (3)
C28	0.0180 (19)	0.0166 (18)	0.0191 (19)	0.0062 (15)	0.0006 (15)	0.0062 (16)
C29	0.0185 (19)	0.024 (2)	0.030 (2)	0.0119 (16)	0.0014 (17)	0.0098 (18)
C30	0.0146 (19)	0.028 (2)	0.038 (3)	0.0102 (16)	0.0064 (17)	0.0109 (19)
C31	0.0158 (19)	0.027 (2)	0.039 (3)	0.0086 (16)	0.0098 (18)	0.0178 (19)
C32	0.0163 (18)	0.0170 (18)	0.024 (2)	0.0076 (15)	0.0060 (15)	0.0078 (16)
C33	0.0153 (18)	0.0181 (18)	0.025 (2)	0.0058 (15)	0.0060 (15)	0.0104 (16)
C34	0.018 (2)	0.037 (2)	0.057 (3)	0.0101 (18)	0.012 (2)	0.031 (2)
C35	0.026 (2)	0.039 (3)	0.088 (4)	0.014 (2)	0.015 (3)	0.046 (3)
C36	0.024 (2)	0.030 (2)	0.066 (4)	0.0137 (19)	0.007 (2)	0.030 (2)
C37	0.0169 (19)	0.0211 (19)	0.032 (2)	0.0099 (15)	0.0048 (17)	0.0128 (18)
Cl1	0.0169 (4)	0.0262 (5)	0.0225 (5)	0.0110 (4)	0.0056 (4)	0.0076 (4)
Ir	0.01303 (7)	0.01454 (7)	0.01389 (7)	0.00593 (5)	0.00301 (5)	0.00519 (5)
N1	0.0135 (15)	0.0195 (16)	0.0182 (16)	0.0080 (13)	0.0019 (12)	0.0006 (13)
N2	0.0202 (17)	0.038 (2)	0.0163 (17)	0.0151 (15)	0.0053 (14)	0.0082 (15)
N3	0.0134 (15)	0.0191 (16)	0.0304 (19)	0.0100 (13)	0.0128 (14)	0.0162 (15)
N4	0.0183 (16)	0.0147 (15)	0.0217 (17)	0.0062 (13)	0.0001 (13)	0.0046 (14)
N5	0.0145 (15)	0.0112 (14)	0.0195 (16)	0.0042 (12)	0.0037 (12)	0.0069 (13)
N6	0.0133 (14)	0.0092 (13)	0.0120 (14)	0.0015 (11)	0.0051 (12)	0.0008 (12)
N7	0.0156 (15)	0.0129 (15)	0.0124 (15)	0.0030 (12)	0.0001 (12)	0.0033 (12)
S1	0.0220 (5)	0.0278 (5)	0.0578 (8)	0.0139 (4)	0.0201 (5)	0.0269 (5)
O1	0.0321 (18)	0.0451 (19)	0.082 (3)	0.0249 (16)	0.0293 (18)	0.048 (2)
O2	0.043 (2)	0.070 (3)	0.125 (4)	0.044 (2)	0.056 (2)	0.077 (3)
O3	0.0299 (17)	0.0252 (15)	0.0365 (18)	0.0082 (13)	0.0119 (14)	0.0133 (14)
C38	0.042 (3)	0.021 (2)	0.066 (4)	0.012 (2)	−0.023 (3)	0.002 (2)
F1	0.081 (2)	0.0412 (17)	0.0464 (19)	0.0292 (17)	−0.0194 (17)	0.0015 (15)
F2	0.071 (3)	0.0259 (16)	0.155 (4)	−0.0036 (16)	−0.066 (3)	0.010 (2)
F3	0.087 (3)	0.067 (2)	0.046 (2)	0.056 (2)	−0.0110 (18)	−0.0086 (17)
S2	0.0451 (15)	0.0461 (18)	0.073 (2)	0.0062 (12)	−0.0005 (13)	0.0441 (17)
O4	0.086 (7)	0.073 (6)	0.058 (6)	0.052 (6)	0.039 (5)	0.053 (5)
O5	0.139 (10)	0.128 (8)	0.072 (3)	0.032 (7)	0.016 (3)	0.041 (3)
O6	0.094 (7)	0.052 (3)	0.216 (11)	−0.004 (2)	−0.028 (7)	0.055 (3)
C39	0.054 (3)	0.072 (4)	0.116 (5)	0.019 (2)	0.018 (3)	0.060 (4)
F4	0.075 (5)	0.102 (5)	0.191 (10)	0.042 (5)	0.027 (6)	0.099 (6)
F5	0.070 (4)	0.078 (4)	0.162 (8)	0.006 (3)	0.016 (4)	0.056 (4)
F6	0.151 (10)	0.180 (10)	0.117 (5)	0.097 (8)	0.032 (3)	0.058 (4)
S2B	0.097 (3)	0.056 (3)	0.0281 (19)	0.031 (2)	0.0234 (19)	0.016 (2)
O4B	0.101 (7)	0.068 (8)	0.040 (4)	0.018 (5)	0.017 (4)	0.030 (4)
O5B	0.157 (11)	0.104 (7)	0.091 (9)	0.041 (7)	0.058 (9)	0.072 (7)
O6B	0.179 (9)	0.067 (4)	0.097 (9)	0.008 (4)	−0.018 (7)	0.018 (4)
C39B	0.128 (6)	0.070 (6)	0.117 (8)	0.050 (5)	0.069 (5)	0.044 (5)
F4B	0.172 (10)	0.157 (14)	0.156 (9)	0.056 (10)	0.036 (8)	0.077 (8)
F5B	0.180 (10)	0.093 (6)	0.174 (11)	0.027 (6)	0.089 (9)	0.009 (6)
F6B	0.149 (10)	0.119 (10)	0.173 (12)	0.070 (8)	0.100 (10)	0.093 (9)

### Geometric parameters (Å, º)

**Table:**

C1—C2	1.389 (6)	C25—H25A	0.9900
C1—N1	1.402 (5)	C25—H25B	0.9900
C1—C6	1.406 (6)	C26—C27	1.529 (6)
C2—C3	1.389 (6)	C26—H26A	0.9900
C2—H2	0.9500	C26—H26B	0.9900
C3—C4	1.404 (7)	C27—H27A	0.9800
C3—H3	0.9500	C27—H27B	0.9800
C4—C5	1.376 (7)	C27—H27C	0.9800
C4—H4	0.9500	C28—N6	1.338 (5)
C5—C6	1.387 (6)	C28—C29	1.391 (5)
C5—H5	0.9500	C28—H28	0.9500
C6—N2	1.387 (6)	C29—C30	1.378 (6)
C7—N1	1.340 (5)	C29—H29	0.9500
C7—N2	1.358 (5)	C30—C31	1.382 (6)
C7—C8	1.472 (5)	C30—H30	0.9500
C8—N3	1.377 (5)	C31—C32	1.389 (5)
C8—C9	1.380 (6)	C31—H31	0.9500
C9—C10	1.391 (6)	C32—N6	1.355 (5)
C9—H9	0.9500	C32—C33	1.471 (5)
C10—C11	1.390 (6)	C33—N7	1.364 (5)
C10—H10	0.9500	C33—C34	1.383 (6)
C11—C12	1.380 (6)	C34—C35	1.378 (6)
C11—H11	0.9500	C34—H34	0.9500
C12—N3	1.347 (5)	C35—C36	1.380 (6)
C12—C13	1.464 (6)	C35—H35	0.9500
C13—N5	1.334 (5)	C36—C37	1.388 (6)
C13—N4	1.365 (5)	C36—H36	0.9500
C14—N4	1.392 (5)	C37—N7	1.341 (5)
C14—C15	1.394 (5)	C37—H37	0.9500
C14—C19	1.413 (5)	Cl1—Ir	2.3510 (9)
C15—C16	1.372 (6)	Ir—N3	1.992 (3)
C15—H15	0.9500	Ir—N1	2.032 (3)
C16—C17	1.409 (6)	Ir—N5	2.037 (3)
C16—H16	0.9500	Ir—N6	2.050 (3)
C17—C18	1.381 (5)	Ir—N7	2.057 (3)
C17—H17	0.9500	S1—O3	1.433 (3)
C18—C19	1.388 (5)	S1—O2	1.444 (3)
C18—H18	0.9500	S1—O1	1.445 (3)
C19—N5	1.392 (5)	S1—C38	1.799 (6)
C20—N2	1.483 (6)	C38—F2	1.327 (6)
C20—C21	1.523 (6)	C38—F1	1.351 (6)
C20—H20A	0.9900	C38—F3	1.355 (6)
C20—H20B	0.9900	S2—O6	1.400 (8)
C21—C22	1.523 (7)	S2—O4	1.430 (8)
C21—H21A	0.9900	S2—O5	1.511 (9)
C21—H21B	0.9900	S2—C39	1.791 (10)
C22—C23	1.524 (7)	C39—F5	1.265 (12)
C22—H22A	0.9900	C39—F4	1.323 (12)
C22—H22B	0.9900	C39—F6	1.425 (13)
C23—H23A	0.9800	S2B—O6B	1.410 (11)
C23—H23B	0.9800	S2B—O4B	1.444 (11)
C23—H23C	0.9800	S2B—O5B	1.508 (10)
C24—N4	1.473 (5)	S2B—C39B	1.818 (12)
C24—C25	1.519 (6)	C39B—F5B	1.300 (15)
C24—H24A	0.9900	C39B—F4B	1.377 (15)
C24—H24B	0.9900	C39B—F6B	1.428 (14)
C25—C26	1.527 (6)		
			
C2—C1—N1	131.4 (4)	H27B—C27—H27C	109.5
C2—C1—C6	121.3 (4)	N6—C28—C29	121.3 (4)
N1—C1—C6	107.3 (4)	N6—C28—H28	119.4
C1—C2—C3	116.6 (4)	C29—C28—H28	119.4
C1—C2—H2	121.7	C30—C29—C28	119.3 (4)
C3—C2—H2	121.7	C30—C29—H29	120.3
C2—C3—C4	121.2 (5)	C28—C29—H29	120.3
C2—C3—H3	119.4	C29—C30—C31	119.1 (4)
C4—C3—H3	119.4	C29—C30—H30	120.4
C5—C4—C3	122.6 (4)	C31—C30—H30	120.4
C5—C4—H4	118.7	C30—C31—C32	119.7 (4)
C3—C4—H4	118.7	C30—C31—H31	120.2
C4—C5—C6	116.1 (4)	C32—C31—H31	120.2
C4—C5—H5	122.0	N6—C32—C31	120.5 (4)
C6—C5—H5	122.0	N6—C32—C33	115.6 (3)
C5—C6—N2	130.5 (4)	C31—C32—C33	123.9 (3)
C5—C6—C1	122.1 (4)	N7—C33—C34	120.9 (4)
N2—C6—C1	107.3 (3)	N7—C33—C32	114.6 (3)
N1—C7—N2	111.9 (3)	C34—C33—C32	124.4 (4)
N1—C7—C8	117.9 (3)	C35—C34—C33	119.3 (4)
N2—C7—C8	130.2 (4)	C35—C34—H34	120.3
N3—C8—C9	119.3 (4)	C33—C34—H34	120.3
N3—C8—C7	110.9 (3)	C34—C35—C36	119.6 (4)
C9—C8—C7	129.7 (4)	C34—C35—H35	120.2
C8—C9—C10	118.4 (4)	C36—C35—H35	120.2
C8—C9—H9	120.8	C35—C36—C37	119.1 (4)
C10—C9—H9	120.8	C35—C36—H36	120.4
C11—C10—C9	121.3 (4)	C37—C36—H36	120.4
C11—C10—H10	119.3	N7—C37—C36	121.4 (4)
C9—C10—H10	119.3	N7—C37—H37	119.3
C12—C11—C10	118.7 (4)	C36—C37—H37	119.3
C12—C11—H11	120.7	N3—Ir—N1	80.34 (13)
C10—C11—H11	120.7	N3—Ir—N5	78.67 (13)
N3—C12—C11	119.8 (4)	N1—Ir—N5	158.99 (13)
N3—C12—C13	108.6 (3)	N3—Ir—N6	99.62 (12)
C11—C12—C13	131.5 (4)	N1—Ir—N6	90.06 (12)
N5—C13—N4	110.9 (3)	N5—Ir—N6	92.58 (11)
N5—C13—C12	119.6 (3)	N3—Ir—N7	178.09 (13)
N4—C13—C12	129.5 (4)	N1—Ir—N7	97.92 (12)
N4—C14—C15	131.5 (3)	N5—Ir—N7	103.06 (12)
N4—C14—C19	107.1 (3)	N6—Ir—N7	79.55 (12)
C15—C14—C19	121.5 (4)	N3—Ir—Cl1	85.00 (9)
C16—C15—C14	116.4 (4)	N1—Ir—Cl1	93.26 (9)
C16—C15—H15	121.8	N5—Ir—Cl1	85.79 (9)
C14—C15—H15	121.8	N6—Ir—Cl1	174.72 (9)
C15—C16—C17	122.5 (4)	N7—Ir—Cl1	95.92 (9)
C15—C16—H16	118.8	C7—N1—C1	106.7 (3)
C17—C16—H16	118.8	C7—N1—Ir	113.3 (2)
C18—C17—C16	121.2 (4)	C1—N1—Ir	139.8 (3)
C18—C17—H17	119.4	C7—N2—C6	106.8 (3)
C16—C17—H17	119.4	C7—N2—C20	128.5 (4)
C17—C18—C19	117.1 (4)	C6—N2—C20	124.5 (3)
C17—C18—H18	121.5	C12—N3—C8	122.4 (3)
C19—C18—H18	121.5	C12—N3—Ir	120.0 (3)
C18—C19—N5	131.9 (4)	C8—N3—Ir	117.2 (3)
C18—C19—C14	121.3 (4)	C13—N4—C14	107.1 (3)
N5—C19—C14	106.8 (3)	C13—N4—C24	127.9 (4)
N2—C20—C21	112.7 (4)	C14—N4—C24	124.9 (3)
N2—C20—H20A	109.1	C13—N5—C19	108.2 (3)
C21—C20—H20A	109.1	C13—N5—Ir	112.6 (3)
N2—C20—H20B	109.1	C19—N5—Ir	138.6 (3)
C21—C20—H20B	109.1	C28—N6—C32	120.1 (3)
H20A—C20—H20B	107.8	C28—N6—Ir	125.0 (2)
C20—C21—C22	113.5 (4)	C32—N6—Ir	114.9 (3)
C20—C21—H21A	108.9	C37—N7—C33	119.5 (3)
C22—C21—H21A	108.9	C37—N7—Ir	124.9 (3)
C20—C21—H21B	108.9	C33—N7—Ir	115.0 (3)
C22—C21—H21B	108.9	O3—S1—O2	114.1 (2)
H21A—C21—H21B	107.7	O3—S1—O1	115.5 (2)
C21—C22—C23	111.0 (5)	O2—S1—O1	115.6 (2)
C21—C22—H22A	109.4	O3—S1—C38	103.1 (2)
C23—C22—H22A	109.4	O2—S1—C38	102.7 (3)
C21—C22—H22B	109.4	O1—S1—C38	103.5 (2)
C23—C22—H22B	109.4	F2—C38—F1	107.6 (4)
H22A—C22—H22B	108.0	F2—C38—F3	106.7 (4)
C22—C23—H23A	109.5	F1—C38—F3	105.3 (5)
C22—C23—H23B	109.5	F2—C38—S1	112.3 (5)
H23A—C23—H23B	109.5	F1—C38—S1	112.8 (3)
C22—C23—H23C	109.5	F3—C38—S1	111.6 (3)
H23A—C23—H23C	109.5	O6—S2—O4	124.0 (7)
H23B—C23—H23C	109.5	O6—S2—O5	111.4 (8)
N4—C24—C25	112.0 (3)	O4—S2—O5	105.0 (7)
N4—C24—H24A	109.2	O6—S2—C39	105.7 (7)
C25—C24—H24A	109.2	O4—S2—C39	106.6 (6)
N4—C24—H24B	109.2	O5—S2—C39	102.0 (6)
C25—C24—H24B	109.2	F5—C39—F4	115.0 (10)
H24A—C24—H24B	107.9	F5—C39—F6	113.3 (11)
C24—C25—C26	113.2 (4)	F4—C39—F6	98.0 (11)
C24—C25—H25A	108.9	F5—C39—S2	114.5 (9)
C26—C25—H25A	108.9	F4—C39—S2	112.8 (8)
C24—C25—H25B	108.9	F6—C39—S2	101.2 (8)
C26—C25—H25B	108.9	O6B—S2B—O4B	114.4 (10)
H25A—C25—H25B	107.8	O6B—S2B—O5B	127.1 (9)
C25—C26—C27	111.6 (4)	O4B—S2B—O5B	112.2 (11)
C25—C26—H26A	109.3	O6B—S2B—C39B	100.0 (9)
C27—C26—H26A	109.3	O4B—S2B—C39B	102.1 (10)
C25—C26—H26B	109.3	O5B—S2B—C39B	93.2 (8)
C27—C26—H26B	109.3	F5B—C39B—F4B	120.8 (16)
H26A—C26—H26B	108.0	F5B—C39B—F6B	113.5 (15)
C26—C27—H27A	109.5	F4B—C39B—F6B	102.2 (14)
C26—C27—H27B	109.5	F5B—C39B—S2B	104.7 (11)
H27A—C27—H27B	109.5	F4B—C39B—S2B	105.3 (12)
C26—C27—H27C	109.5	F6B—C39B—S2B	109.9 (10)
H27A—C27—H27C	109.5		
			
N1—C1—C2—C3	179.6 (4)	C1—C6—N2—C20	−175.4 (4)
C6—C1—C2—C3	−0.7 (6)	C21—C20—N2—C7	−72.8 (5)
C1—C2—C3—C4	0.9 (6)	C21—C20—N2—C6	101.0 (5)
C2—C3—C4—C5	−0.8 (7)	C11—C12—N3—C8	0.9 (6)
C3—C4—C5—C6	0.4 (7)	C13—C12—N3—C8	−177.1 (3)
C4—C5—C6—N2	179.6 (4)	C11—C12—N3—Ir	173.3 (3)
C4—C5—C6—C1	−0.2 (6)	C13—C12—N3—Ir	−4.7 (4)
C2—C1—C6—C5	0.4 (6)	C9—C8—N3—C12	0.1 (6)
N1—C1—C6—C5	−179.9 (4)	C7—C8—N3—C12	177.4 (3)
C2—C1—C6—N2	−179.5 (4)	C9—C8—N3—Ir	−172.5 (3)
N1—C1—C6—N2	0.3 (4)	C7—C8—N3—Ir	4.8 (4)
N1—C7—C8—N3	−6.6 (5)	N5—C13—N4—C14	−0.2 (4)
N2—C7—C8—N3	174.4 (4)	C12—C13—N4—C14	−178.4 (4)
N1—C7—C8—C9	170.4 (4)	N5—C13—N4—C24	175.7 (3)
N2—C7—C8—C9	−8.6 (7)	C12—C13—N4—C24	−2.5 (7)
N3—C8—C9—C10	−0.2 (6)	C15—C14—N4—C13	179.7 (4)
C7—C8—C9—C10	−177.0 (4)	C19—C14—N4—C13	−0.1 (4)
C8—C9—C10—C11	−0.6 (7)	C15—C14—N4—C24	3.6 (7)
C9—C10—C11—C12	1.5 (7)	C19—C14—N4—C24	−176.2 (3)
C10—C11—C12—N3	−1.7 (6)	C25—C24—N4—C13	−79.1 (5)
C10—C11—C12—C13	175.7 (4)	C25—C24—N4—C14	96.1 (4)
N3—C12—C13—N5	−1.1 (5)	N4—C13—N5—C19	0.5 (4)
C11—C12—C13—N5	−178.7 (4)	C12—C13—N5—C19	178.8 (3)
N3—C12—C13—N4	177.0 (4)	N4—C13—N5—Ir	−172.5 (2)
C11—C12—C13—N4	−0.7 (7)	C12—C13—N5—Ir	5.9 (4)
N4—C14—C15—C16	−179.6 (4)	C18—C19—N5—C13	−179.8 (4)
C19—C14—C15—C16	0.2 (6)	C14—C19—N5—C13	−0.5 (4)
C14—C15—C16—C17	−0.3 (6)	C18—C19—N5—Ir	−9.7 (7)
C15—C16—C17—C18	0.3 (6)	C14—C19—N5—Ir	169.6 (3)
C16—C17—C18—C19	−0.1 (6)	C29—C28—N6—C32	1.5 (5)
C17—C18—C19—N5	179.2 (4)	C29—C28—N6—Ir	−176.1 (3)
C17—C18—C19—C14	0.0 (6)	C31—C32—N6—C28	−2.0 (5)
N4—C14—C19—C18	179.8 (3)	C33—C32—N6—C28	175.6 (3)
C15—C14—C19—C18	0.0 (6)	C31—C32—N6—Ir	175.8 (3)
N4—C14—C19—N5	0.4 (4)	C33—C32—N6—Ir	−6.6 (4)
C15—C14—C19—N5	−179.4 (3)	C36—C37—N7—C33	1.0 (6)
N2—C20—C21—C22	−57.6 (5)	C36—C37—N7—Ir	−170.0 (3)
C20—C21—C22—C23	−172.8 (5)	C34—C33—N7—C37	0.8 (6)
N4—C24—C25—C26	−69.9 (5)	C32—C33—N7—C37	−176.3 (3)
C24—C25—C26—C27	−179.0 (4)	C34—C33—N7—Ir	172.7 (3)
N6—C28—C29—C30	0.2 (6)	C32—C33—N7—Ir	−4.4 (4)
C28—C29—C30—C31	−1.4 (6)	O3—S1—C38—F2	−179.1 (3)
C29—C30—C31—C32	0.9 (6)	O2—S1—C38—F2	−60.4 (4)
C30—C31—C32—N6	0.8 (6)	O1—S1—C38—F2	60.2 (4)
C30—C31—C32—C33	−176.6 (4)	O3—S1—C38—F1	−57.3 (4)
N6—C32—C33—N7	7.3 (5)	O2—S1—C38—F1	61.5 (4)
C31—C32—C33—N7	−175.2 (4)	O1—S1—C38—F1	−177.9 (4)
N6—C32—C33—C34	−169.7 (4)	O3—S1—C38—F3	61.1 (4)
C31—C32—C33—C34	7.8 (7)	O2—S1—C38—F3	179.9 (4)
N7—C33—C34—C35	−1.9 (7)	O1—S1—C38—F3	−59.5 (4)
C32—C33—C34—C35	174.9 (5)	O6—S2—C39—F5	169.9 (11)
C33—C34—C35—C36	1.1 (8)	O4—S2—C39—F5	−56.5 (12)
C34—C35—C36—C37	0.7 (8)	O5—S2—C39—F5	53.4 (11)
C35—C36—C37—N7	−1.8 (7)	O6—S2—C39—F4	35.9 (13)
N2—C7—N1—C1	−0.3 (4)	O4—S2—C39—F4	169.5 (11)
C8—C7—N1—C1	−179.5 (3)	O5—S2—C39—F4	−80.7 (12)
N2—C7—N1—Ir	−175.6 (2)	O6—S2—C39—F6	−67.9 (10)
C8—C7—N1—Ir	5.2 (4)	O4—S2—C39—F6	65.7 (10)
C2—C1—N1—C7	179.7 (4)	O5—S2—C39—F6	175.6 (9)
C6—C1—N1—C7	0.0 (4)	O6B—S2B—C39B—F5B	172.2 (13)
C2—C1—N1—Ir	−6.9 (7)	O4B—S2B—C39B—F5B	54.4 (15)
C6—C1—N1—Ir	173.4 (3)	O5B—S2B—C39B—F5B	−59.1 (14)
N1—C7—N2—C6	0.5 (4)	O6B—S2B—C39B—F4B	−59.4 (14)
C8—C7—N2—C6	179.6 (4)	O4B—S2B—C39B—F4B	−177.2 (14)
N1—C7—N2—C20	175.1 (4)	O5B—S2B—C39B—F4B	69.2 (14)
C8—C7—N2—C20	−5.8 (7)	O6B—S2B—C39B—F6B	50.0 (15)
C5—C6—N2—C7	179.7 (4)	O4B—S2B—C39B—F6B	−67.8 (16)
C1—C6—N2—C7	−0.4 (4)	O5B—S2B—C39B—F6B	178.7 (15)
C5—C6—N2—C20	4.8 (7)		

### Hydrogen-bond geometry (Å, º)

**Table:**

*D*—H···*A*	*D*—H	H···*A*	*D*···*A*	*D*—H···*A*
C5—H5···Cl1^i^	0.95	2.74	3.422 (4)	130
C9—H9···O5^ii^	0.95	2.42	3.084 (11)	126
C9—H9···O5*B*^ii^	0.95	2.19	3.052 (13)	151
C20—H20*B*···O6^ii^	0.99	2.48	3.259 (13)	135
C20—H20*B*···O5*B*^ii^	0.99	2.52	3.406 (13)	149
C24—H24*B*···O3^iii^	0.99	2.46	3.419 (5)	163
C25—H25*A*···F2^iv^	0.99	2.56	3.287 (5)	131
C28—H28···O4	0.95	2.19	3.063 (11)	152
C28—H28···O4*B*	0.95	2.34	3.196 (18)	150
C31—H31···O2^v^	0.95	2.45	3.380 (5)	165
C34—H34···O2^v^	0.95	2.35	3.298 (5)	177
C36—H36···O3^vi^	0.95	2.45	3.333 (5)	155
C37—H37···O1^vi^	0.95	2.49	3.302 (5)	144

Symmetry codes: (i) −*x*+1, −*y*, −*z*; (ii) −*x*+1, −*y*+1, −*z*; (iii) −*x*+1, −*y*+1, −*z*+1; (iv) *x*+1, *y*+1, *z*; (v) −*x*, −*y*, −*z*+1; (vi) −*x*+1, −*y*, −*z*+1.
